# Royal London Space Planning as a learning tool for postgraduate Orthodontic students. A mixed methods study

**DOI:** 10.1177/14653125221119219

**Published:** 2022-08-17

**Authors:** Farooq Ahmed, Pratik K Sharma

**Affiliations:** 1 Guy’s & St Thomas’ NHS Foundation Trust; 2 Centre for Oral Bioengineering, Barts & The London School of Medicine and Dentistry, QMUL, Whitechapel, UK

**Keywords:** orthodontic education, space analysis, specialty training, Royal London Space Planning, treatment planning

## Abstract

**Objective:**

To assess the effectiveness of the Royal London Space Planning (RLSP) as a learning tool among postgraduate orthodontic students as well as investigate students’ attitudes to its use. The RLSP tool is a structured method of orthodontic case assessment and treatment planning.

**Design:**

A prospective cohort study of first year postgraduate orthodontic students who attended teaching of the RLSP.

**Setting:**

Postgraduate teaching institute.

**Participants:**

First year postgraduate orthodontic students.

**Methods:**

The outcome measured was the accuracy in assessment and planning of a standardised orthodontic simulated case before and after teaching. Qualitative assessment was conducted through focus group and a semi-structured format after the teaching.

**Results:**

Nineteen students were included in the study. There was an overall improvement in assessment and planning of 20% after the teaching intervention (*P* < 0.05). Assessment improved by 34% in comparison to treatment planning, which improved by 17% (*P* < 0.05). The impact of the RLSP was most noticeable on the assessment of crowding in the lower arch which improved by 37% after teaching (*P* < 0.05). Students felt using the RLSP tool made them more confident and was advantageous to use in training; most felt they would not use the tool after qualification.

**Conclusion:**

The RLSP tool is an effective method of teaching assessment and treatment planning to postgraduate orthodontic students. The effect of the RLSP is greater at assessments and less significant for treatment planning. The participant students felt the RLSP improved their confidence in assessment and treatment planning.

## Background

Postgraduate orthodontic training is the most common postgraduate dental specialty training pathway in the UK with 155 trainees. The syllabus from the Specialist Advisory Committee in Orthodontics (SAC) of the General Dental Council (GDC) includes specialist-specific objectives relating to orthodontic assessment and diagnosis. Trainees are expected to conduct a systematic and thorough space analysis and evaluate the information for the purposes of treatment planning (The Specialist Advisory Committee in Orthodontics, 2010).

Space analysis is defined as the quantification of space required in each arch for the correction of malocclusion (Proffit et al., 2006). Different applications of space analysis have been proposed and can be divided into three categories: mixed dentition analysis (Proffit et al., 2006); unerupted dentition analysis (Hixon and Oldfather, 1958; Moyers, 1973; Tanaka and Johnston, 1974) and permanent dentition analysis (Kirschen et al., 2000a; Proffit et al., 2006). Mixed dentition space analysis pertains to interceptive treatment planning, unerupted dentition analysis relates to planning of unerupted teeth, and permanent space analysis relates to definitive treatment planning. The focus of this study was definitive treatment planning.

Two main forms of permanent dentition analysis have been described. One method presented by Proffit et al. (2006) consists of measuring the arch perimeter and mesial distal widths of the dentition. However, Proffit et al.’s assessment relates to crowding only and does not factor other features of assessment incumbent within space planning, such as incisor retraction, expansion and so on. The applicability of this assessment is limited to selective malocclusions. The second method of The Royal London Space Planning (RLSP), which has two components, is one of a structured method of space analysis (Table 1) and the second component relating to treatment planning using the information from the former (Table 2) (Kirschen et al., 2000a, 2000b). The RLSP is used for teaching postgraduate orthodontic students within Queen Mary University of London and is a part of the Greater London Orthodontic Training Programme. The advantages of the RLSP tool are to allow detailed evaluation of the components of orthodontic treatment; it facilitates the evaluation of space utilisation during treatment, the feasibility of treatment goals evaluated, treatment mechanics can be determined together with demands on anchorage, and an understanding of outcomes of treatment to be achieved.

**Table 1.** Royal London Space Analysis (Assessment).

	Lower	Upper
Crowding and spacing:	________mm	________mm
Levelling occlusal curve:	________mm	________mm
Arch width change:	________mm	________mm
Incisor A/P change:	________mm	________mm
Angulation/inclination change:	________mm	________mm
	Total ________mm	________mm

**Table 2.** Royal London Space Planning (Treatment planning).

Tooth reduction/enlargement: (+ or −) extractions:	________mm	________mm
Space opening for prosthetic replacement:	+________mm	+________mm
Molar distal movement:	−________mm	−________mm
Molar mesial movement:	+________mm	+________mm
Differential U/L growth: (+ or −)	−________mm	−________mm

The effectiveness of the RLSP as a teaching tool for postgraduates in orthodontics has not been investigated. The aim of this study was to assess the effectiveness of the RLSP on postgraduate students as a learning tool for space analysis and treatment planning. The attitudes of participants to this tool were also assessed.

## Materials and methods

The investigation was undertaken through a prospective cohort study design. Funding and ethical approval was obtained from the Queen Mary Ethics of Research Committee (QMREC1838a). The sample consisted of first year postgraduate students in orthodontics in the UK who had no previous postgraduate space planning teaching and were attending the Greater London Orthodontic Programme. The intervention was teaching of the RLSP delivered as a one-day, face-to-face, structured teaching session with a practical component. The outcome measures were quantitative and qualitative.

### Simulated orthodontic case assessment and planning (quantitative measure)

Quantitative assessment was undertaken through a simulated assessment of a standardised clinical case in an in-classroom setting. Students completed a bespoke questionnaire capturing the student’s assessment and treatment planning answers. The questionnaire was completed three weeks before the RLSP teaching intervention. The same clinical case and questionnaire were repeated two weeks after the interventional teaching. Trainees were advised to make quantitative measurements to within 0.5 mm.

**Figure 1.**
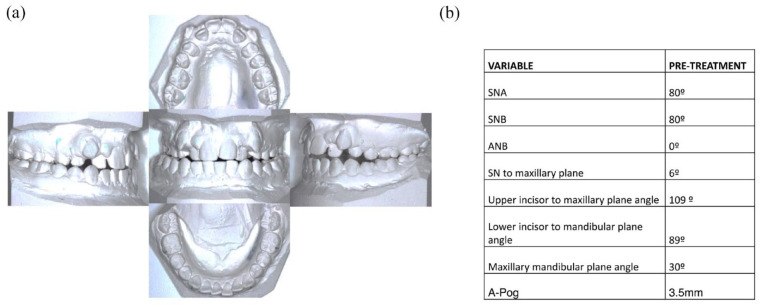
(a) Simulated orthodontic case models. (b) Simulated orthodontic case cephalometric values.

A simulated orthodontic case was used in the study and verified by two senior consultants experienced in application and teaching of the RLSP to ensure the case incorporated all six elements of the tool. The case used consisted of a 14-year-old patient in their permanent dentition, with a class III incisor relationship with a skeletal III pattern, reduced overjet and overbite with maxillary and mandibular crowding (Figure 1). Participants were provided with the following information relating to the simulated orthodontic case: case introduction; intra-oral photographs; 1:1 scaled photos of clinical models; and cephalometric values (Figure 1). Participants were given 30 min to assess the case and complete the questionnaire.

The questionnaire assessing the participants’ analysis and planning was derived from the RLSP diagnosis and treatment planning components (Kirschen et al., 2000a, 2000b). Nine questions were formed with sub-questions that were deemed appropriate. Answers were either multiple choice options, with the most appropriate answer to be selected, or free-text answers. The questionnaire was piloted on second year postgraduate orthodontic trainees (who had previously undergone RLSP teaching). Questions were assessed for face validity, and feedback from the pilot questionnaire was used to modify the final questionnaire. Where providing potential answers was found to be leading, a free-text section was used to capture the response.

Investigator blinding was achieved through participant’s questionnaires being pseudo- anonymised. This was necessary to correlate pre-interventional education questionnaires with post-interventional questionnaires. A code was generated for each pre-interventional questionnaire and identified to the participant’s name. The same occurred for the post-interventional questionnaire. Participant-sensitive data were matched with the pseudo-anonymous code and stored on an encrypted computer, following coding of before and after questionnaires; participant sensitive data were not accessed.

### Focus group (qualitative measure)

**Figure 2.**
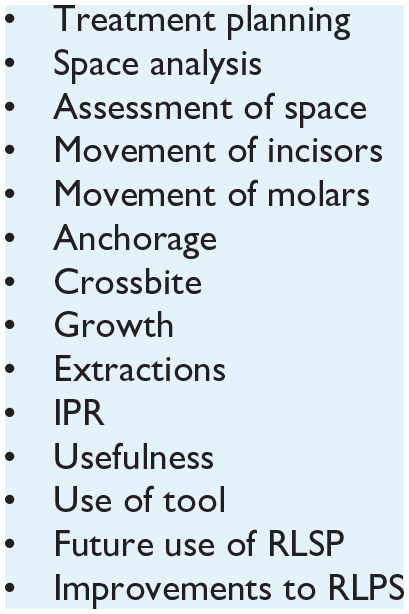
Topic guide for focus group.

After the RLSP teaching, a focus group comprising five participants was undertaken in a semi-structured format. Topic guides were used to aid development of the conversation (Figure 2). The topic guide was developed by the research team of key questions to be asked in order to explore areas related to the research objectives. Notes were taken in the focus group by the interviewer (FA). The focus group was recorded and transcribed by FA. A framework approach to analysis was adapted from Ritchie et al. (2003), which included identification of themes from the transcriptions, labelling the data, sorting data by theme, data synthesis with thematic charts, and discussion of the themes within the research group.

### Statistical analysis

Data were coded and analysed using Excel version 15.18 (Microsoft Inc., Redmond, WA, USA). Descriptive analysis was carried out via frequency analysis where appropriate. McNemar’s test was used where possible to compare responses to each question before and after teaching; significance was set at *P* < 0.05. Analysis was conducted per protocol.

## Results

**Figure 3.**
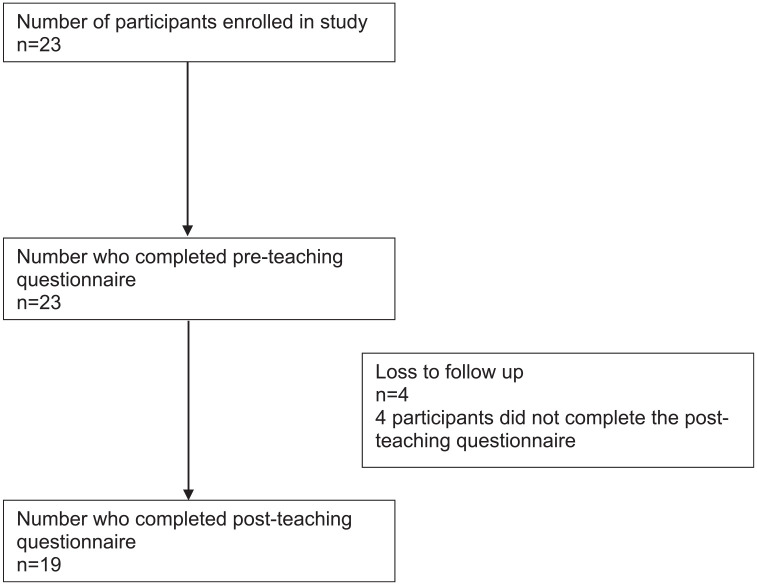
Participant flow diagram.

A total of 23 orthodontic trainees were approached to complete the questionnaire and 19 completed and matched responses were achieved (Figure 3). Four trainees failed to attend the second session.

The results of each question are presented below with pre- and post-interventional teaching data (Table 3).

*Question 1: What was the total arch crowding?*

The question was subdivided into upper and lower arches. In the upper arch, before RLSP teaching, correct responses were 68%. After RLSP teaching, participants scored 100%. In the lower arch, before RLSP teaching, 58% had the correct response, with 95% correct responses after RLSP teaching. There was an improvement in assessing crowding in the upper arch by 32% and in the lower arch by 37%.

*Question 2: How would you treat this case?*

The clinical case was deemed to be amenable to ‘camouflage’. Before RLSP teaching, 79% of trainees agreed. After RLSP teaching, 95% of trainees chose camouflage as an appropriate method of managing the clinical case, showing an improvement in 16%.

*Question 3: Do you plan to advance the maxillary incisors?*
The correct answer was to ‘maintain maxillary incisors’. Before and after RLSP teaching scores were unchanged, with only 11% of trainees agreeing with the ideal treatment standard.
*Question 4: Do you plan to retract the mandibular incisors?*
The correct answer was to ‘retract the mandibular incisors’. Before RLSP teaching, 58% of trainees agreed with the statement; this increased by 31% after RLSP teaching to 89%. A sub-question enquired the quantity of mandibular incisor retraction and the reasoning; there was an improvement of 30% after the RLSP teaching.
*Question 5: Do you plan to correct the posterior crossbite?*
The correct answer was ‘yes’ to correct the crossbite. All participants (100%) answered correctly before and after RLSP teaching. Regarding a sub-question enquiring which method would be used to achieve transverse correction (expand upper arch, constrict lower arch or both), RLSP teaching improved correct responses by 47%.
*Question 6: What (if any) influence would growth have on your space planning?*

The correct answer was ‘no influence’ in the upper arch and ‘mandibular advancement’ in the lower arch. In the upper arch, before RLSP teaching, 26% of participants answered the question correctly; after RLSP teaching, 47% answered correctly, demonstrating a 21% improvement. In the lower arch, a similar improvement of 21% was observed.

*Question 7: How do you plan to create the space needed in the upper arch?*

‘Through dental extractions’ was the correct answer. Before RLSP teaching, 42% scored correctly and after RLSP teaching, 68% answered correctly, showing an improvement of 26%. A sub-question asked which teeth would be removed; the answer was ‘either upper 1st or 2nd premolars’. After RLSP teaching, there was an improvement in 31% in the correct upper arch dental extraction pattern. A further sub-question asked about how much mesial movement of the first molars was expected; after RLSP teaching, there was an improvement of 27%.

*Question 8: How do you plan to create the space needed in the lower arch?*

The correct answer was also ‘extractions’. Before RLSP teaching, 26% of participants answered correctly; after teaching, 53% answered correctly. There was an increase in 27% after teaching.

*Question 9: Will you be reinforcing anchorage?*

The correct answer was ‘yes’. Before RLSP teaching, 68% of participants answered correctly; after RLSP teaching, 84% answered correctly, showing an increase of 16%. A sub-question asked what type of anchorage would be used; the correct answer was either ‘inter-arch elastics’, ‘TPA’ or ‘nance’. Before RLSP teaching, 27% of participants answered correctly; after RLSP teaching, 32% answered correctly.

**Table 3.** Questions 1–9.

	Correct answers before teaching	Correct answers after teaching	*P* value
Question 1: What was the total arch crowding?			
Upper arch	13 (68)	19 (100)	0.03*
Lower arch	11 (58)	18 (95)	0.02*
Question 2: How would you treat this case?			
	15 (79)	18 (95)	0.25
Question 3: Do you plan to advance the maxillary incisors?			
	2 (11)	2 (11)	N/A
Question 4: Do you plan to retract the mandibular incisors?			
	11 (58)	17 (89)	0.03*
Question 5: Do you plan to correct the posterior crossbite?			
	19 (100)	19 (100)	1*
Question 6: What (if any) influence would growth have on your space planning?			
Upper arch	5 (26)	9 (47)	0.13
Lower arch	7 (37)	11 (58)	0.13
Question 7: How do you plan to create the space needed in the upper arch?			
	8 (42)	13 (68)	0.06
Question 8 How do you plan to create the space needed in the lower arch?			
	5 (26)	10 (53)	0.06
Question 9: Will you be reinforcing anchorage?			
	13 (68)	16 (84)	0.25
Combined overall results	102 (54)	141 (74)	<0.0001*

Values are given as n (%).

* Statistically significant.

### Focus group results

Focus group assessment revealed consistent themes of trainee experiences with the RLSP tool. Four reoccurring themes emerged.

#### Advantages of using the tool

Participants felt the RLSP made them ‘quicker and able to get a better outcome’. Participants also felt ‘more confident after the teaching’ when it came to treatment planning.

#### Future use of the RLSP

Participants felt it would be useful to continue using the tool during training and would be ‘thorough with it’; however, the majority of the focus group felt they would not use the tool formally after qualification and the tool would be used for ‘tricky cases’ or informally.

#### Disadvantaged orthodontic participants who do not use the RLSP

Participants commented on the difficulty in learning assessment and treatment planning if they were not taught the RLSP. Participants concluded that without the structured RLSP trainees would be at a disadvantage in assessment and treatment planning.

#### Improvements of the RLSP

Participants felt the RLSP could be improved through clearer guidance on the ‘influence of growth’, better understanding of ‘interproximal reduction’, ‘inclusion of asymmetries’ and clearer guidance on when to change and maintain ‘incisor inclination’.

## Discussion

This study was the first assessment of the structured RLSP tool for the purpose of dental education. Overall, the results show a significant increase in space analysis assessment and treatment planning accuracy through RLSP teaching (Table 3).

The RLSP had a positive effect on the assessment and treatment planning of postgraduate orthodontic students by 20%. There was a greater effect on assessment and a lesser effect on treatment planning (Table 4), with a 34% improvement in assessment and only a 17% improvement in treatment planning. This may be due to assessment being an accuracy skill, whereas treatment planning requires a combination of skills, such as deduction, reasoning and fore planning, skills which are time- and experience-dependent.

**Table 4.** Combined assessment and combined treatment planning results.

Combined questions	Correct answers before teaching	Correct answers after teaching	*P* value
Assessment (1)	24 (63)	37 (97)	<0.0001*
Treatment planning (2–9)	78 (51)	104 (68)	<0.0001*

Values are given as n (%).

* Statistically significant.

The most significant finding is an increase in 34% of accuracy of crowding assessment of participants using a clear ruler. Different methods of assessing crowding have been described. Johal and Battagel (1997) investigated the reflex microscope, visual assessment using a clear ruler and the brass wire technique. They concluded all three methods yielded similar precision; however, reflex microscopy was an expensive resource (Johal and Battagel, 1997). No literature has been published evaluating the assessment of crowding accuracy with teaching tools.

**Figure 4.**
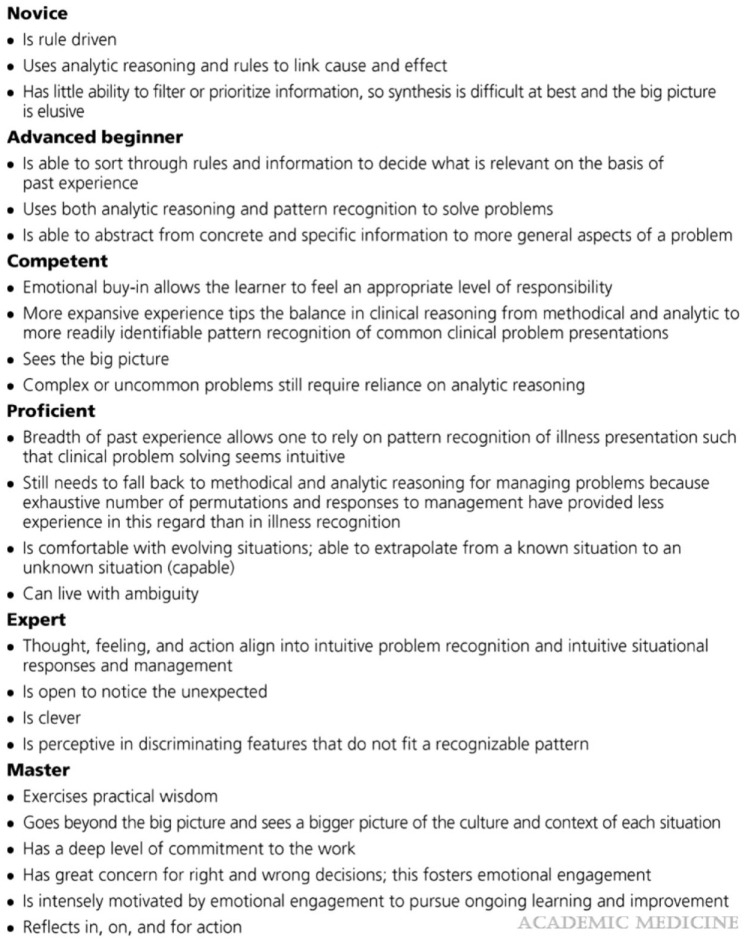
Modified Drayfus and Drayfus model (Carraccio et al., 2008).

Learning the theory of a new skill has been described in medicine as the modified Drayfus and Drayfus model (Figure 4) by Carraccio et al. (2008). The key components relevant to the learning of orthodontic assessment and treatment planning are as follows: theoretical knowledge; organisation of ideas; experience; intellect; and a reflective process. The RLSP tool design facilitates learners achieving the first two components, theoretical knowledge and organisation of ideas. The aim of moving learners from ‘novice’ to ‘competent’ can be achieved in part through the RLSP tool.

Use of a structured assessment tool has been shown to increase clinical effectiveness in performing a clinical skill as well as delivering more consistent and comprehensive communication to patients (Marshall et al., 2009). The Health Research Authority’s advice on good practice for consent advises on the use of tools which aid understanding of the diagnosis and the outcomes of treatment (Health Research Authority). With recent changes to consenting processes from Bolam to Montgomery, practitioners are required to communicate outcomes and risks of the proposed treatment and alternative treatments (1957) (UK Supreme Court, 2015). The RLSP allows practitioners to quantify and predict occlusal outcomes and therefore qualify justification of a proposed treatment plan.

In clinical practice, the RLSP has been shown to have limited application. Al-Abdullah et al. (2008) assessed the RLSP for reliability and influence on treatment planning through the case assessment of 31 patient records by 17 examiners. Good reliability was shown with intra-examiner reliability (intraclass correlation coefficient [ICC] = 0.77–0.93) and for inter-examiner reliability (ICC = 0.88–0.93). However, the influence on treatment planning was moderate at 0.52 (ICC = 0.24–0.82). They concluded that the RLSP tool gave greater diagnostic yield but did not influence treatment planning when compared to not using the tool, thus did not offer any advantage in treatment planning (Al-Abdallah et al., 2008). This study involved trainees and consultants, with varying degrees of treatment planning skills, which may have affected the validity of the treatment planning results. In this study, it has been shown that the RLSP also has a lesser effect on developing treatment planning skills when compared to developing assessment skills for trainees.

The RLSP has been assessed for sensitivity and specificity through case assessments of 30 untreated patient records by Dause et al. (2010). They concluded that the RLSP had good sensitivity for assessment of crowding and spacing; however, it had poor specificity. They concluded that the RLSP was deficient in treatment planning due to weaknesses in planning for arch asymmetries and the effect of growth (Dause et al., 2010). The sample was selected from a historic database with the possibility of sampling bias present.

Qualitative analysis revealed the RLSP tool improved students’ confidence in assessment and planning. The use of structured compared to unstructured education in medicine has shown improved confidence in perception of education from students, as well as an increase in positive outlook on the educational topic (Van der Hem-Stokroos et al., 2003). Interestingly, students felt the structured approach was advantageous during training years and without it students would be disadvantaged; however, the majority felt they would not use the tool beyond qualification. Perhaps this was due to students perceiving that after qualifying they would move from trying to achieve a competent state of analysis and planning, to proficient and expert where pattern recognition and intuition are used (Carraccio et al., 2008).

Our findings, together with those outlined above, suggest that the RLSP is an effective tool for space analysis and treatment planning when used in education and learning of orthodontics. The use of such skills will prepare future orthodontists in a technologically advancing field, where both commercial drive and automation can potentially influence assessment and treatment planning.

## Limitations

The present study has some limitations. The sample selected was of a small sample of participants and was a sample of convenience. A more robust methodology would have been to conduct a randomised control trial with one arm receiving the RSLP teaching and the other not receiving teaching. Greater assessment of the learning could have been conducted through several case assessments and treatment planning to show greater reliability of the learning. However, due to the small number of postgraduate orthodontic students and the logistical challenges of arranging training across different sites, a single sample from the Greater London Orthodontic Programme was used to reduce confounding factors. The assessment was limited to one clinical case to not fatigue the participants. Further assessment was not possible due to the participants attending from a variety of institutions where further formal and informal learning on space analysis and treatment planning would have proved to be another confounding factor.

## Conclusion

In conclusion, the RLSP tool is an effective method of teaching assessment and treatment planning to postgraduate orthodontic students. Second, the effect of the RLSP is greater for assessments and less significant for treatment planning. Finally, the participating students felt that the RLSP improved their confidence in assessment and treatment planning.
